# A community intervention to reduce alcohol consumption and drunkenness among adolescents in Sweden: a quasi-experiment

**DOI:** 10.1186/s12889-021-10755-3

**Published:** 2021-04-21

**Authors:** Robert Svensson, Björn Johnson, Karl Kronkvist

**Affiliations:** 1 Department of Criminology, Malmö University, 205 06 Malmö, Sweden; 2 Department of Social Work, Malmö University, 205 06 Malmö, Sweden

**Keywords:** Adolescents, Alcohol use, Intervention, Prevention, The Öckerö method

## Abstract

**Background:**

Several studies have examined the effect of community interventions on youth alcohol consumption, and the results have often been mixed. The aim of this study is to evaluate the effectiveness of a community intervention known as the Öckerö Method on adolescent alcohol consumption and perceived parental attitudes towards adolescent drinking.

**Method:**

The study is based on a quasi-experimental design, using matched controls. Self-report studies were conducted among adolescents in grades 7–9 of compulsory education in four control and four intervention communities in the south of Sweden in 2016–2018. Baseline measures were collected in autumn 2016 before the intervention was implemented in the intervention communities. Outcomes were the adolescents’ alcohol consumption, past-year drunkenness, past-month drunkenness and perceived parental attitudes towards alcohol.

**Results:**

Estimating Difference-in-Difference models using Linear Probability Models, we found no empirical evidence that the intervention has any effect on adolescents’ drinking habits, or on their perceptions of their parents’ attitudes towards adolescent drinking.

**Conclusion:**

This is the first evaluation of this method, and we found no evidence that the intervention had any effect on the level of either young people’s alcohol consumption or their past-year or past-month drunkenness, nor on their parents’ perceived attitudes toward adolescent drinking. A further improvement would be to employ a follow-up period that is longer than the three-year period employed in this study.

**Trial registration:**

ISRCTN registry: Study ID: 51635778, 31th March 2021 (Retrospectively registered).

**Supplementary Information:**

The online version contains supplementary material available at 10.1186/s12889-021-10755-3.

## Background

Alcohol use among young people is a risky behavior that is associated with several negative consequences, including an increased risk for accidents, health-related problems and involvement in crime [1–5]. The risk of developing alcohol dependence later in life is also greater for individuals with an early alcohol debut [2, 6]. It is therefore important to introduce effective prevention strategies to reduce the use of alcohol and other drugs among young people.

While there are many methods to achieve this aim, one approach involves the use of whole-of-community interventions. This approach typically targets a well-defined population within a delimited geographical region and includes the implementation of several simultaneously coordinated interventions across various community settings (e.g., schools, sports clubs, social services, law enforcement, etc.) [7, 8]. By activating multiple community stakeholders in the intervention process, the ultimate goal for these types of interventions is, as with many other types of interventions, to delay the onset of alcohol use among adolescents and decrease their general alcohol consumption during adolescence [8]. While the whole-of-community approach usually includes a multi-component strategy to achieve these goals, e.g., by focusing on demand-, harm- and supply-reduction strategies [8], one common and arguably important component relates to altering social norms and attitudes related to drinking [9, 10].

Several studies have examined the effect of community interventions on youth alcohol consumption across different international contexts, including for example Australia [9], Canada [11], Iceland [12], the Netherlands [13], Sweden [14], and the USA [15]. Here, a strong focus has been directed towards changing social norms and attitudes relating to underage drinking among adolescents [9, 11, 14]. Strategies has included youth social skills training [11, 14], information and communication with both youths, parents and other community actors regarding risks and harms with underage drinking [9, 11, 13, 14], mass media campaigns to increase public awareness of issues relating to underage drinking [9, 13–15], and youth access to alcohol [14, 15]. In some of these studies it has been found that the intervention had an impact on youth alcohol consumption [12, 13], whereas other studies found none or only minor impact [11, 14, 15]. According to systematic reviews and meta-analyses the rather mixed findings in previous research could be a consequence of specific components included in these interventions not being evidence-based [8, 16]. Another shortcoming discussed in relation to community interventions often involve methodological problems, with local organizations initiating the intervention, for example, leaving researchers with no other options than ex post facto research designs [8, 13]. Despite a growing number of studies on the effect of community interventions on youth alcohol consumption, the current knowledge base would benefit from additional research using robust methodological designs, including prospective evaluations.

One community intervention which has recently received attention in Sweden is the Öckerö Method. This is a community-based alcohol and drug prevention method which was developed in Sweden at the beginning of the 2000s and is used in approximately 30 municipalities in Sweden and Finland. The method is designed for use in relatively small communities and may be described as employing a whole-of-community approach with the goal of delaying the onset of alcohol use and reducing alcohol consumption among youths by strengthening restrictive attitudes and approaches to youth alcohol consumption among parents and other adults.

The aim of this study is to evaluate the effectiveness of the Öckerö Method. More specifically, we will focus on two research questions: (1) Is it possible to identify effects of the Öckerö Method on youths’ alcohol consumption and drunkenness? (2) Is it possible to identify effects of the Öckerö Method on parental attitudes towards alcohol consumption and drunkenness, based on the youths’ perceptions?

## Method

### The intervention

The Öckerö Method is a community intervention that aims to change social norms of adolescents with regards to alcohol consumption, by providing information to parents, other adults, local associations and local media, with the intent of influencing their attitudes towards alcohol consumption by adolescents. The method is implemented by local prevention workers, and is followed up by means of self-report surveys that are conducted once each year with adolescents in secondary school. The results from these surveys are reported to parents, school staff, social services administrations and the public. The results are also employed continuously in the work conducted in the intervention municipalities. In this way, parents, responsible agencies and other local actors are given continuous information on the alcohol and drug situation among the municipality’s secondary school youth.

The different activities used in the intervention are presented in detail below:

*Information at school parent meetings in grades 7, 8 and 9*. Information is provided at parents’ meetings once per year, directly subsequent to the completion of the self-report survey. The information provided at these parents’ meetings includes (1) up to date information on alcohol, tobacco and drug use among students in the municipality (e.g., the prevalence of alcohol consumption and drunkenness, what types of beverages the adolescents usually drink and how they get hold of the alcohol) (2) information on risks associated with youth drinking in the form of risks for immediate (acute) harms, risks for long-term harms (e.g., that an early alcohol debut is associated with an increased risk of developing alcohol problems), and the links between alcohol and other drugs, and (3) advice to parents on what they can do to prevent and discourage their children from drinking alcohol. Examples of such advice include (a) talk to your adolescent about alcohol, establish restrictive rules and ensure that the adolescent knows that these rules are protective measures, (b) be informed about your adolescent’s whereabouts, activities, and friendships, (c) prepare your adolescent for peer pressure (d) make sure that one parent is always awake if the adolescent comes home late. The parents are also informed that it is possible to teach their adolescents how to drink responsibly without allowing them to drink. Finally, the parents are encouraged to talk to the other class-parents and make it clear that they want to be contacted if another parent notices that their adolescent has been drinking.

*Newsletters to parents and other adults*. Newsletters are sent via e-mail 3–6 times per year to parents and to other adults who register to receive them. These newsletters present results and more in-depth analyses from the self-report surveys, and other forms of up to date information on the alcohol and drug situation among youths in the municipality, for example information in connection with public holidays and similar occasions that may be associated with partying among young people. The information contained in the newsletters is also communicated via the Facebook accounts of the local drug prevention coordinators.

*Information work directed at the local community*. Information is provided to sports clubs and other associations in the local community, mainly through meetings with youth leaders. The objective here is to influence these clubs and associations to ensure that their youth activities are always alcohol free. The contacts with clubs and associations also involve providing information from the local drug self-report survey, similar to the information provided at parent meetings in schools.

*Information via local media*. Information is also disseminated via local news media outlets. This information is normally disseminated in connection with the annual self-report surveys, but can also be communicated at other times, for example in connection with public holidays. The main aim of this work is to implement public health education campaigns about the harms of risky drinking via local media outlets. Press releases to local radio stations and local newspapers include information on the local alcohol and drug situation among youths, and on local prevention work.

For a summary of the interventions see Table 1.

**Table 1:** Summary of interventions

	2016	2017	2018	2019
Baseline – self-report survey: August—September	x			
Annual self-report survey: August—September		x	x	x
*Interventions:*				
a. Information at parents’ meetings (directly after the self-report survey)	x	x	x	x
b. Newsletter to parents (3–6 times per year, beginning after the self-report survey)	x	x	x	x
c. Informational work directed at the local community	x	x	x	x
d. Public health message via local media (after the self-report survey)	x	x	x	x

### Design, sample and matching

To examine the effect of the Öckerö Method, this study employs a prospective quasi-experimental research design. Eight small municipalities located in the southern region of Sweden (Skåne) have been sampled. Skåne was chosen as the evaluation area in part because the Öckerö Method has not previously been implemented in Skåne, but also because the level of alcohol consumption among youth in Skåne lies approximately 10–20% above the national average for Sweden [17]. The eight municipalities were included in the sample because, according to the regional coordinators for drug and alcohol prevention at the County Administration Board, they needed to develop their drug prevention work and were not working with any of the components included in the Öckerö Method.

The sampled municipalities were paired on the basis of a number of matching variables, including average school results, average educational level within the municipality, the economic situation of households within the municipality and the proportion of municipal residents of non-Swedish background. One municipality in each pair was then randomly assigned to either intervention (Öckerö Method) or control conditions (treatment as usual).

Data on the adolescents’ alcohol use and parental attitudes towards alcohol have been collected anonymously by means of an annual self-report survey among the approximately 3500 secondary school students who are enrolled each year in the municipalities’ schools (7th through 9th grade). These students are distributed across 17 schools in the eight municipalities included in the study (nine in the intervention municipalities, eight in the control municipalities). The data collection was administered by project assistants who visited the schools in the participating municipalities once a year during the period 2016–2019 at the beginning of the autumn term. The baseline survey was conducted between August 17 and September 1, 2016, and the follow-up surveys were conducted during the same period during the years 2017–2019.

Since the study was conducted in the entire secondary schools (grades 7–9) each year, it is possible to use different study designs. In the current study we will conduct longitudinal comparisons of the intervention and control groups, following a cohort of youths who were in grade 7 in 2016 during the period 2016—2018 (from grade 7 to grade 9). A sensitivity analysis is also conducted for the subsequent grade 7 cohort, i.e. youths who were in grade 7 in 2017, who are followed until 2019 (when they were in grade 9).

The level of external missing data was 10–12% per year, giving a total of 12,486 completed questionnaires. This represents a response rate of 88.2%, with no marked difference between the intervention and control municipalities. In this article we analyze data from the first cohort, i.e. those who were in grade 7 in 2016, a group comprising approximately 1000 participants per year, giving us a total of 3035 observations for the period 2016–2018. Adolescents who moved to the municipalities during the study period were identified through a screening question and were excluded from the analyses. The sample is presented in more detail in Table 2. For a flow diagram of the participants and inclusion, see Supplemental appendix.

**Table 2:** Participants in control and intervention groups

	Autumn 2016 Baseline (grade 7)	Autumn 2017 Time 1 (grade 8)	Autumn 2018 Time 2 (grade 9)
*Control:*			
Sample:	575	595	648
Included in the analyses:	534	497	527
Non-response:	7.1%	16.5%	18.7%
*Intervention:*			
Sample:	543	534	571
Included in the analyses:	519	474	484
Non-response:	4.4%	11.2%	15.2%

### Ethical considerations

According to the Act concerning the Ethical Review of Research Involving Humans (Act 2003:460), parents must be informed and must consent to research that includes children under the age of 15. The study is based on the passive consent of the parents, i.e. we informed the parents that their children would be invited to participate in the study, and asked those parents who did not want their children to participate to inform us of this via e-mail, post or telephone. A non-response on the part of the parents was interpreted as indicating consent.^1^ All students in grades 7–9 (aged 13–15 at the start of the autumn term) were informed about the study both verbally and in writing prior to the initiation of the data collection process. Among the students themselves, the study is based on active consent, with the participating students showing their consent by completing and sending in the questionnaire. The study has been assessed and approved by the Regional Ethics Review Board in Lund (application no. 2016/88).

### Measures

#### Primary outcome: drinking

Three measures of drinking will be used in this study. *Alcohol consumption* is measured using the following item: “Have you ever drunk alcohol (by alcohol we mean medium-strength or strong beer, cider, alcopop, wine or spirits)?” Response options: no (0), yes, 1 time (1), and yes, many times (2). We have dichotomized the variable in the following way: no or yes, one time (0) and yes, many times (1). *Drunkenness past year* is measured using the following item: “How many times during the past 12 months have you drunk alcohol so that you have felt intoxicated?” Response options: never (0) and 1 time or more (1). *Drunkenness past month* is measured using the following item: “How many times during the past month have you drunk alcohol so that you have felt intoxicated?” Response options: never (0) and 1 time or more (1).

#### Secondary outcome: parental attitudes towards alcohol use as perceived by the adolescents

*Perceived parental attitudes towards alcohol* are measured by an additive index comprised of two items: For my parents, it’s okay (1) if I drink alcohol (2) if I get drunk. Response options: neither agree nor disagree, somewhat agree and strongly agree (0) and strongly disagree and somewhat disagree (1). The correlation between the two dichotomized items varies between *r* = .42 and *r* = .54 over the 3 years. We combined the two dichotomized items into a single item (with a range of: 0–2). Then this variable was dichotomized in the following way: 0–1 is coded as (0) and 2 is coded as (1), were the latter indicates restrictive attitudes towards alcohol.

#### Control variables

*Gender* is coded 0 for girls and 1 for boys. *Born in Sweden* is coded as 0 if the respondent is born abroad and 1 if the respondent is born in Sweden. *Split family* is coded as 0 if the respondent is living with both biological parents and 1 if this is not the case.

### Statistical analyses

First, we compare differences between the control and intervention groups with regard to our control variables, using chi-square tests. Secondly, we compare differences between control and intervention groups in relation to our primary outcome (drinking variables) and our secondary outcome (perceived parental attitudes) across the 3 years. Finally, to examine whether there is an intervention effect we estimate a Difference-in-Difference (DD) model. The DD model is a quasi-experimental design and have been used in studies with similar designs as the present study [18], and more widely in public health policy research when randomized controlled trials are not applicable [19]. The basic idea behind the DD model is to compare the change in any given outcome in an intervention group before and after a hypothesized intervention is introduced while accounting for any concurrent change in a control group not receiving that particular intervention [19–21].

As the outcome variables are binary, we decided to estimate our model using Linear Probability Model (LPM) [22, 23]. The LPM is described in eq. (1), where *Y*_*it*_ represents the four different outcome variables: alcohol consumption, past-year drunkenness, past-month drunkenness and parental attitudes toward alcohol for adolescent *i* in grade *t*:

1 $$ {Y}_{it}={\beta}_0+{\beta}_1{Intervention}_i+{\beta}_2{Post}_t+{\beta}_3\left({Intervention}_i\times {Post}_t\right)+{\beta}_4{X}_{it}+{\varepsilon}_{it} $$

*Intervention* is a dummy variable indicating whether an adolescent attends an intervention community school (equal to 1 if so, otherwise 0). *Post (time)* is a dummy variable for post-treatment data (equal to 1 for grade 8 or 9, and equal to 0 for grade 7). The interaction term *Intervention* × *Post* is the causal effect of the method on the outcome variables, and *β*_3_ is the coefficient of main interest. This variable will indicate whether there are any differences in the outcome variables between the intervention and control groups, i.e. this is our measure of the effect of the intervention. Finally, *X*_*it*_ is a vector of individual-specific control variables and *ε*_*it*_ is the error term.

The models have been estimated in the following stages. Two DD models were estimated for each of our four outcome variables. In Model 1 we included the intervention variable, the post variable and the intervention × post interaction variable. In Model 2 we adjusted for our three control variables, gender, born in Sweden and split family. As the data are based on respondents who are clustered in schools, robust clustered standard errors are presented for the LPM models. The use of robust clustered standard errors takes account of the fact that the observations may be correlated within schools.^2^ All analyses were conducted in Stata/SE version 13.1.

One assumption for the DD analysis is that the intervention and control groups follow a common trend in relation to the studied outcome prior to the intervention [21]. Since the current study only includes one pre-intervention time period, we are not able to examine pre-intervention trends among adolescents in either of the groups. However, since intervention and control municipalities have been matched pairwise, based on a number of relevant variables, and were then randomly assigned to intervention or control conditions, we have no reason to suspect different pre-intervention trends. Comparisons between control and intervention groups at baseline for control variables (i.e. gender, ethnicity and living in a split family) are made to assess the possibility of different trajectories in the two groups prior to the intervention.

## Results

### Descriptive characteristics

Table 3 presents descriptive statistics for the control variables over baseline, Time 1 and Time 2. The results for baseline show no significant differences in control variables between the intervention and control groups for gender (51.9% cf. 49.9% boys), birthplace (88.7% cf. 88.6% born in Sweden), and family structure (29.1% cf. 28.5% living in a split family) for the baseline. The results are stable over Time 1 and Time 2.

**Table 3:** Descriptive statistics for the control variables

	Baseline (grade 7)		Time 1 (grade 8)		Time 2 (grade 9)		Total
	Control	Intervention	Control	Intervention	Control	Intervention	
*Gender:*							
Girls (%)	48.1	50.1	45.7	49.5	49.8	49.7	48.8
Boys (%)	51.9	49.9	54.3	50.5	50.2	50.3	51.2
Total	532	517	492	469	526	483	3019
*Born in Sweden:*							
No (%)	11.3	11.4	14.7	14.8	18.9	16.4	14.5
Yes (%)	88.7	88.6	85.3	85.2	81.1	83.6	85.5
Total	533	517	496	472	525	482	3025
*Split family:*							
No (%)	70.9	71.5	70.8	71.8	69.3	71.4	70.9
Yes (%)	29.1	28.5	29.2	28.2	30.7	28.6	29.1
Total	530	516	490	471	525	479	3011

Note. No significant differences in control variables between the control and intervention groups were found at baseline, Time 1 or Time 2

### Programme effects on drinking

**Fig. 1:**
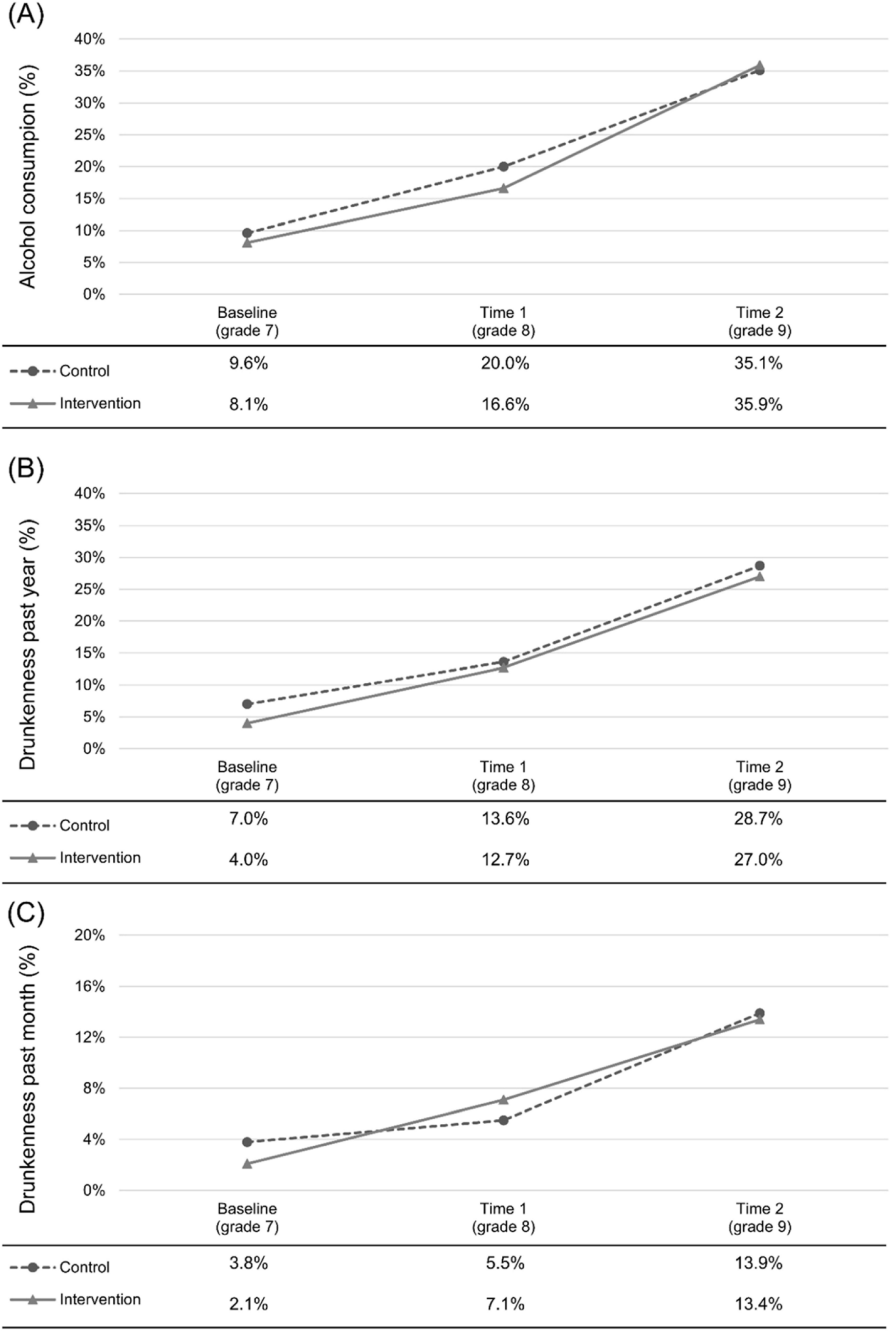
Trend in proportion reporting alcohol consumption (**a**), past-year drunkenness (**b**), and past-month drunkenness (**c**) in the control and intervention groups

Figure 1 presents the three-year trend for alcohol consumption, past-year and past-month drunkenness for control and intervention groups. At the baseline, 9.6% of the adolescents in the control group reported alcohol consumption, compared with 8.1% for the intervention group. Thereafter there was an increase in alcohol consumption at time 1 and time 2, with a similar gradient across the control and intervention groups. For past-year and past-month drunkenness, the control and intervention groups reported drunkenness on a similar level at baseline. In the following 2 years, the proportion of adolescents reporting that they had been drunk during the past-year and past-month was also similar for the control group and the intervention group.

Results from the DD model are presented in Table 4. In the first model, our intervention variable is not significantly associated with our drinking measures. The results also show that our post variable is significant and positively associated with all three drinking measures. This indicates that our three measures of alcohol use increase over time and that this increase is significant for both the control and the intervention group. Finally, our interaction term intervention × post is not significantly associated with our drinking measures. This indicates that the program has not had any significant effect over time. All these results are stable after adjusting for gender, being born in Sweden and living in a split family, as shown in Model 2.

**Table 4:** Difference-in-Difference models, LPM estimates for drinking

	Alcohol consumption				Drunkenness past year				Drunkenness past month			
	Model 1		Model 2		Model 1		Model 2		Model 1		Model 2	
	Coef.	*p-*value	Coef.	*p-*value	Coef.	*p-*value	Coef.	*p-*value	Coef.	*p-*value	Coef.	*p-*value
Intervention	−.015	.344	−.016	.306	−.029	.127	−.031	.108	−.016	.151	−.017	.134
Post	.182	<.001	.184	<.001	.144	<.001	.144	<.001	.061	<.001	.059	<.001
Intervention × Post	.000	.995	.001	.988	.015	.703	.016	.689	.021	.285	.020	.282
Boy			−.026	.236			−.047	.019			−.035	.021
Born in Sweden			.053	.044			.021	.441			.000	.997
Split family			.072	.003			.075	.003			.028	.020
*R*^2^	.046		.056		.041		.055		.017		.023	
N	3018		2972		3015		2969		3012		2966	

Note: The *p*-values are calculated using robust standard errors, clustered by schools

### Programme effects on parental attitudes toward alcohol use as perceived by their adolescents

**Fig. 2:**
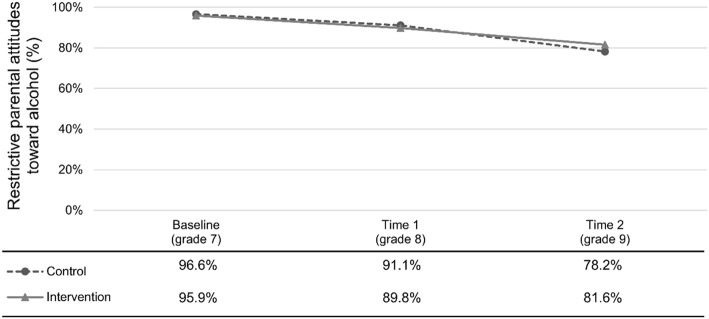
Trend in proportion of parents with restrictive attitudes towards alcohol use as perceived by the adolescents in the control and intervention groups

Figure 2 shows the trend for perceived parental attitudes towards alcohol use, as reported by the adolescents. The results show that the baseline, i.e. grade 7, level of parents who thought it was not okay to use alcohol and to be drunk was very high, after which we can see a decrease over time. This means that the older the children become, the higher the proportion of parents with a less restrictive approach to drinking. The pattern is similar for both the control and the intervention group for the two first years, whereas the lines separate somewhat between time 1 and time 2.

Finally, Table 5 presents the results from the DD model focused on parental attitudes towards alcohol use. In the first model, only our post variable is significant and positively associated with parental attitudes towards alcohol use. This indicates that parents become less restrictive about alcohol use over time and that this change is significant for both the control and the intervention group. The interaction term intervention × post is not significantly associated with parental attitudes towards alcohol. This indicates that the program has not had any effect on the parents’ attitudes over time. The results are stable after adjusting for the three control variables in Model 2.

**Table 5:** Difference-in-Difference models, LPM estimates for parental attitudes towards alcohol use as perceived by the adolescents

	Model 1		Model 2	
	Coef.	*p-*value	Coef.	*p-*value
Intervention	−.007	.420	−.005	.604
Post	−.121	<.001	−.123	<.001
Intervention × Post	.019	.574	.017	.608
Boy			.030	.123
Born in Sweden			−.037	.109
Split family			−.029	.135
*R*^2^	.029		.036	
N	2995		2950	

Note: The *p*-values are calculated using robust standard errors, clustered by schools

### Sensitivity analyses

We ran a number of alternative models in order to test the robustness of our findings. First, we repeated our series of models using logistic regression and focusing on estimates of marginal effects. The results followed a pattern similar to that obtained using LPM. Second, we estimated our LPM models for girls and boys, with the results showing no indication of an effect of the interaction term. Third, analyses have also been estimated using the second wave cohort, i.e. those adolescents who started grade 7 in 2017 and went on to grade 9 in 2019. In these additional models, we treated the year 2017 as the baseline. Although this group may have been affected by the intervention to some extent at baseline, we wanted to examine whether the results are also stable in relation to this cohort. The results show no indication of an intervention effect. Fourth, we also estimated comparisons between baseline vs. Time 1, baseline vs. Time 2 and Time 1 vs. Time 2, and the results are the same as those presented in Tables 3 and 4. Fifth, the project’s research design also allowed us to compare the intervention and control groups by year group, i.e. following the trends within each grade (7, 8, and 9) over a period of 4 years, 2016–2019. The trends were very similar for all grades.

## Discussion

The aim of this study was to conduct an independent evaluation of the effectiveness of the community intervention known as the Öckerö Method. Using a prospective quasi-experimental design, we examined two questions: First, is it possible to identify effects on youths’ alcohol consumption and drunkenness? Second, is it possible to identify effects on parental attitudes towards alcohol consumption and drunkenness, based on the youths’ perceptions?

The empirical results from our Difference-in-Difference analyses are clear. We found no evidence that the intervention had any effect on the level of either young people’s alcohol consumption or their past-year or past-month drunkenness, nor on their parents’ perceived attitudes toward adolescent drinking. A number of sensitivity models were also estimated, producing stable results; no significant effect of the program was found. Our finding of no effects is in line with the results of a number of previous studies that have been unable to identify effects of community interventions in relation to alcohol use [10, 18, 26], although some other studies have found empirical support for an effect of broader community interventions in relation to alcohol use [12, 13, 27].

There could be *two* possible interpretations for these results. First, the method does not have the expected effect (by comparison with municipalities that have not implemented the method), or that the method is not sufficient to produce such an effect, i.e., some parts of the method may work while other parts are not effective, or it is possible that none of the various interventions included in the method produce an effect. Since we lack dose-response measures, however, we are unable to say which of these is the case in this study. Second, the follow-up period may be too short, and the effect of the intervention may not become measurable until later. The importance of having an observation period that is sufficiently long has been discussed in the research literature, particularly in relation to community interventions [13, 28].

Although this study employs a well-founded research design with a large-scale sample and low levels of non-response, there are a few limitations that need to be addressed. Firstly, this is a quasi-experimental study which lacks randomization at the school and individual level (RCT). Although a number of community interventions studies are based on quasi-experimental designs [6, 8], further evaluations of the Öckerö Method need to use an RCT design, such as the cluster RCT for example [8, 29]. Secondly, in this study we do not examine how different components of the community intervention work in isolation; that would be something for further research to examine. Thirdly, parental attitudes are described by the youths, whereas other studies have also included data collected from the parents themselves [30, 31].

## Conclusion

This is the first evaluation of this method, and we have found no evidence that the intervention had any effect on the level of either young people’s alcohol consumption or their past-year or past-month drunkenness, nor on their parents’ perceived attitudes toward adolescent drinking. A possible improvement would be to employ a follow-up period that is longer than the three-year period employed in this study. Finally, the different components of the method need to be revised, and thereafter, more systematic and formal evaluations are needed.

## Supplementary Information

**Additional file 1.**

**Additional file 2.**

## Data Availability

The datasets used in the current study are not publicly available due to restrictions made by the Regional Ethical Review Board in Lund, Sweden, but are available from the corresponding author on reasonable request.

## Abbreviations

DD: Difference-in-Difference
LPM: Linear Probability Model
AME: Average marginal effects
RCT: Randomized controlled trial

## Footnotes

1 A total of 15 parents contacted the researchers to say that their children would not be participating in the study.

2 Since the number of clusters are rather few [21, 24] we also estimated the models using cluster bootstrap standard errors that gives the same results as the one published in the results section [24, 25].
