# Supplementary material for: A community intervention to reduce alcohol consumption and drunkenness among adolescents in Sweden: a quasi-experiment
Source: BMC Public Health. 2021 Apr 21;21:764. doi: 10.1186/s12889-021-10755-3 (PMC8058986; doi:10.1186/s12889-021-10755-3)
Supplement: Supplementary file 1 — Additional file 1. [file 12889_2021_10755_MOESM1_ESM.docx]

**Enrollment**

Municipalities referred to intervention (n=8)

Pairwise randomization (n=8)

Pairwise matching

Allocated to intervention:

Öckerö Method (n=4)

- Students enrolled in grade 7 (n=543)
- Students in grade 7 completing survey (n=519; 95.6%)

**Allocation**

- Students enrolled in grade 8 (n=534)
- Students in grade 8 completing survey (n=492; 92.1%)

**Time 1**

**Baseline**

- Students enrolled in grade 9 (n=530)
- Students in grade 9 completing survey (n=500; 94.3%)

Included in analyses:

- Baseline (n=519)
- Time 1 (n=474)
- Time 2 (n=484)

**Analysis**

**Time 2**

Allocated to control:

Treatment as usual (n=4)

Secondary schools (n=9)

Excluded from analyses:

- Not attending school in same municipality previous year (n=77)
- Students enrolled in grade 7 (n=575)
- Students in grade 7 completing survey (n=534; 92.9%)
- Students enrolled in grade 8 (n=595)
- Students in grade 8 completing survey (n=517; 86.9%)
- Students enrolled in grade 9 (n=620)
- Students in grade 9 completing survey (n=550; 88.7%)

Included in analyses:

- Baseline (n=534)
- Time 1 (n=497)
- Time 2 (n=527)

Secondary schools (n=8)

Flow diagram for the study
