# Supplementary material for: A community intervention to reduce alcohol consumption and drunkenness among adolescents in Sweden: a quasi-experiment
Source: BMC Public Health. 2021 Apr 21;21:764. doi: 10.1186/s12889-021-10755-3 (PMC8058986; doi:10.1186/s12889-021-10755-3)
Supplement: Supplementary file 2 — Additional file 2. [file 12889_2021_10755_MOESM2_ESM.docx]

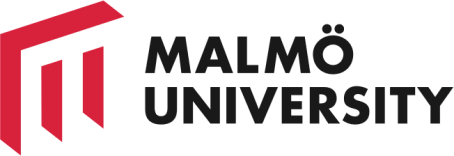


**Would you like to participate in a survey about alcohol and drug habits among secondary school students?**

The aim of the survey is to examine the effects of the Öckerö method, an alcohol and drug prevention method. The survey is directed at all students in years 7, 8 and 9 in your municipality and in several other municipalities in Skåne.

In this questionnaire we ask questions about you, your family and your friends. We also ask about whether you like being in school and what you do in your leisure time. There are also a number of questions about alcohol, tobacco and drugs.

Participating in the survey is voluntary. You can decline to participate if you want to, or you can stop filling in the questionnaire at any time without giving a reason and without this having any negative consequences for you.

Everyone who answers the questionnaire will be anonymous. This means that nobody knows who you are or what you have answered. No school staff, parents or anyone else will see your answers.

Answer the questions in order by putting a cross in the box ☒ for the answer that is most true for you. Sometimes you will be able to put a cross in more than one box, when it says to do so.

When you have completed the questionnaire and checked that you haven’t missed any questions, you put the questionnaire in the envelope and seal it. People working in the project will collect the envelopes.

If you do not want to participate in the survey, you just put the blank questionnaire in the envelope without filling it in. In this way, nobody knows which students have chosen to participate or not.

The project is being conducted at Malmö University. The research leader is Björn Johnson, professor in social work. Robert Svensson, professor of criminology, is also working in the project.

Contact details:

Björn Johnson, e-mail: bjorn.johnson@mah.se, telephone: 040-665 76 90

Robert Svensson, e-mail: robert.svensson@mah.se, telephone: 040-665 77 81

*The survey begins on the next page!*

| ***First some questions about you and your family*** |
| --- |

**1. What school year are you in?**

□ Year 7 □ Year 8 □ Year 9

__________________________________________________________________________________

**2. Are you a girl or a boy?** □ Girl □ Boy

__________________________________________________________________________________

**3. Which municipality do you live in?**

□ Bjuv □ Hörby □ Klippan □ Osby

□ Simrishamn □ Skurup □ Tomelilla □ Östra Göinge

__________________________________________________________________________________

**3 (b). Which school do you attend?**

□ Allé School

□ Ekeby School

□ Frosta School

□ Jens Bille School

□ Georgshills School

□ Göinge School

□ Hasslaröds School

□ Kastanje School

□ Korsavads School

□ Ljungbyheds School

□ Ludvigsborgs Free School

□ Macklean School

□ New Snyggatorps School

□ Park School

□ Snapphane School

□ Varagårds School

□ Örkened School

**4. Where were you born?**

□ In Sweden

□ In Denmark, Finland, Iceland or Norway

□ In another country in Europe

□ In a country outside Europe

__________________________________________________________________________________

**5. How long have you lived in Sweden?**

□ All my life

□ Between 0 and 2 years

□ Between 2 and 5 years

□ More than 5 years

__________________________________________________________________________________

**6. Where were your parents born? Mother Father**

In Sweden □ □

In Denmark, Finland, Iceland or Norway □ □

In another country in Europe □ □

In a country outside Europe □ □

__________________________________________________________________________________

**7. What do your parents do? Mother Father**

Work □ □

Is studying □ □

Leave of absence/parental leave □ □

Unemployed/jobseeker □ □

Retired □ □

Don’t know □ □

__________________________________________________________________________________

**8. Do you live together with your mother and your father?**

□ Yes

□ Only with my father

□ Only with my mother

□ Sometimes with my mother, sometimes with my father

□ I don’t live with either my mother or my father

**9. Do you live with a stepparent?**

□ No □ Yes, with my stepfather □ Yes, with my stepmother

__________________________________________________________________________________

**10. Do you have any siblings?**

□ No

□ Yes, older

□ Yes, younger

□ Yes, both older and younger

__________________________________________________________________________________

**11. Put a cross in the box that is most true for you!**

|  | No,  never | rarely | sometimes | often | Yes,  always |
| --- | --- | --- | --- | --- | --- |
| Do you have fixed times when you have to be home in the evenings? | □ | □ | □ | □ | □ |
| Do you have to ask your parents’ permission to go out in the evening? | □ | □ | □ | □ | □ |
| Do you have to contact your parents if you can’t come home by a certain time? | □ | □ | □ | □ | □ |
| If you are going out in the evening, do you have to tell your parents whom you will be meeting? | □ | □ | □ | □ | □ |
| If you are going out in the evening, do you have to tell your parents what you are going to do? | □ | □ | □ | □ | □ |

**12. There now follow some questions about your relationship with your parents. If you feel you have better contacts with your stepparents, you can answer about them instead, if you want to.**

|  | No,  never | rarely | sometimes | often | Yes,  Always |
| --- | --- | --- | --- | --- | --- |
| Do you feel that your mother trusts you? | □ | □ | □ | □ | □ |
| Do you feel that your father trusts you? | □ | □ | □ | □ | □ |
| Do you usually feel that your mother gives you support and encouragement? | □ | □ | □ | □ | □ |
| Do you usually feel that your father gives you support and encouragement? | □ | □ | □ | □ | □ |
| Do you think you have a good contact with your mother? | □ | □ | □ | □ | □ |
| Do you think you have a good contact with your father? | □ | □ | □ | □ | □ |
| Do you feel that your mother cares about you? | □ | □ | □ | □ | □ |
| Do you feel that your father cares about you? | □ | □ | □ | □ | □ |
| Can you usually talk about everything (e.g. problems) with your mother? | □ | □ | □ | □ | □ |
| Can you usually talk about everything (e.g. problems) with your father? | □ | □ | □ | □ | □ |

| *There now follow some questions about school, your leisure time and your friends* |
| --- |

**13. Put a cross in the box to show whether the following statements about school are true for you**

**!**

|  | Completely untrue | Fairly untrue | Neither true nor untrue | Fairly true | Completely true |
| --- | --- | --- | --- | --- | --- |
| I really enjoy school. | □ | □ | □ | □ | □ |
| I get on well with my teachers. | □ | □ | □ | □ | □ |
| I care what my teachers think about me. | □ | □ | □ | □ | □ |
| I do my best at school. | □ | □ | □ | □ | □ |
| I would definitely leave school immediately if this were possible. | □ | □ | □ | □ | □ |
| If I have homework, I do it straight away. | □ | □ | □ | □ | □ |

**14. Do you play truant?**

□ No

□ Yes, about once a term

□ Yes, about once a month

□ Yes, 2-3 times a month

□ Yes, once a week

□ Yes, several times a week

__________________________________________________________________________________

**15. Have you been bullied during the past 12 months?**

□ No

□ Once or twice

□ Yes, 2-3 times a month

□ Yes, about once a week

□ Yes, several times a week

**16. Have you participated in bullying someone during the past 12 months?**

□ No

□ Once or twice

□ Yes, 2-3 times a month

□ Yes, about once a week

□ Yes, several times a week

__________________________________________________________________________________

**17. How many evenings a week do you usually meet friends?**

| □ 0 | □ 1 | □ 2 | □ 3 | □ 4 | □ 5 | □ 6 | □ 7 |
| --- | --- | --- | --- | --- | --- | --- | --- |

__________________________________________________________________________________

**18. How many evenings a week do you usually go out to the shopping centre, or into town on your own or with friends?**

| □ 0 | □ 1 | □ 2 | □ 3 | □ 4 | □ 5 | □ 6 | □ 7 |
| --- | --- | --- | --- | --- | --- | --- | --- |

__________________________________________________________________________________

**19.** **How many days a week do you usually just hang out or drift around without doing anything in particular, either on your own or with friends?**

| □ 0 | □ 1 | □ 2 | □ 3 | □ 4 | □ 5 | □ 6 | □ 7 |
| --- | --- | --- | --- | --- | --- | --- | --- |

__________________________________________________________________________________

**20. How many days a week do you usually participate in sports activities (e.g. dance, football, going to the gym, horse-riding, swimming)**

| □ 0 | □ 1 | □ 2 | □ 3 | □ 4 | □ 5 | □ 6 | □ 7 |
| --- | --- | --- | --- | --- | --- | --- | --- |

__________________________________________________________________________________

**21. How many days a week do you usually go to a youth club?**

| □ 0 | □ 1 | □ 2 | □ 3 | □ 4 | □ 5 | □ 6 | □ 7 |
| --- | --- | --- | --- | --- | --- | --- | --- |

__________________________________________________________________________________

**22. How many days a week do you usually have music lessons or go to a choir?**

| □ 0 | □ 1 | □ 2 | □ 3 | □ 4 | □ 5 | □ 6 | □ 7 |
| --- | --- | --- | --- | --- | --- | --- | --- |

__________________________________________________________________________________

**23. How many days a week do you usually do something with your parents?**

| □ 0 |  | □ 1 | □ 2 | □ 3 | □ 4 | □ 5 | □ 6 | □ 7 |
| --- | --- | --- | --- | --- | --- | --- | --- | --- |

**24. How often do you use a computer, mobile phone or tablet for the following activities?**

|  | Every day | Several times a week | About once a week | About once a month | Never |
| --- | --- | --- | --- | --- | --- |
| Talk to friends on Kik, Viber, What's App or similar | □ | □ | □ | □ | □ |
| Stay in touch with and stay informed about my friends via Facebook or similar | □ | □ | □ | □ | □ |
| Post information about myself on Facebook, Instagram, Snapchat or other social media | □ | □ | □ | □ | □ |
| Play games | □ | □ | □ | □ | □ |
| Listen to music | □ | □ | □ | □ | □ |
| Visit websites to learn about one of my interests | □ | □ | □ | □ | □ |
| Share files containing music, films or video clips | □ | □ | □ | □ | □ |
| Watch films or video clips | □ | □ | □ | □ | □ |
| Post my own music or films on sites such as Instagram, YouTube | □ | □ | □ | □ | □ |
| Read daily newspapers or check the news | □ | □ | □ | □ | □ |

**25. Have any of your closest friends done any of the following (that you know about) during the past month?**

|  | No, no friends | Yes, 1 friend | Yes, 2-3 friends | Yes, more than 3 friends |
| --- | --- | --- | --- | --- |
| Taken something from a shop without paying? | □ | □ | □ | □ |
| Destroyed (vandalised) something? | □ | □ | □ | □ |
| Knocked someone down? | □ | □ | □ | □ |
| Drunk to the point of becoming intoxicated? | □ | □ | □ | □ |
| Used marijuana, hash or other drugs? | □ | □ | □ | □ |

**26. Mark with a cross whether the following statements about your closest friends are true or not for you.**

|  | Completely untrue | | Fairly untrue | Fairly true | Completely true |
| --- | --- | --- | --- | --- | --- |
| I can trust my friends. | | □ | □ | □ | □ |
| My friends mean a lot to me. | | □ | □ | □ | □ |
| I can talk about all kinds of problems with my friends. | | □ | □ | □ | □ |
| My friends care about me. | | □ | □ | □ | □ |
| My friends are always there for me if I need help with anything. | | □ | □ | □ | □ |

| **27. The friends I most like to spend time with think it is completely okay to…** | Completely untrue | | Fairly untrue | Fairly true | Completely true |
| --- | --- | --- | --- | --- | --- |
| …drink a beer at the weekend | | □ | □ | □ | □ |
| …drink to the point of becoming intoxicated | | □ | □ | □ | □ |
| …smoke cigarettes | | □ | □ | □ | □ |
| …use marijuana or hash | | □ | □ | □ | □ |

**28. How wrong do you think it is for someone of**

**your age to …**

|  | Very  wrong | wrong | A bit wrong | Not wrong at all |
| --- | --- | --- | --- | --- |
| …tease a classmate about their clothes? | □ | □ | □ | □ |
| …steal something from a shop? | □ | □ | □ | □ |
| …smoke cigarettes? | □ | □ | □ | □ |
| …drink a beer at the weekend? | □ | □ | □ | □ |
| …drink to the point of becoming intoxicated? | □ | □ | □ | □ |
| … use marijuana or hash? | □ | □ | □ | □ |
| …hit someone so that he/she gets hurt? | □ | □ | □ | □ |

| *There now follow some questions on alcohol, tobacco and drugs* |
| --- |

**29. Have you ever drunk alcohol? (By alcohol we mean medium-strength beer, strong beer, strong cider, alcopop, wines or spirits.)**

□ No ***If you have answered no, go to question 30***

□ Yes, 1 time

□ Yes, several times

__________________________________________________________________________________

**29 (b). How many times during the past 12 months have you drunk alcohol so that you felt intoxicated?**

| □ Never | □ 1 time | □ 2-3 times | □ 4-5 times | □ 6-10 times | □ More than 10 times |
| --- | --- | --- | --- | --- | --- |

__________________________________________________________________________________

**29 (c). How many times during the past four weeks have you drunk alcohol so that you felt intoxicated?**

| Never | 1  Time | 2  times | 3  times | 4  times | 5  Times | 6  times | 7 times or more |
| --- | --- | --- | --- | --- | --- | --- | --- |
| □ | □ | □ | □ | □ | □ | □ | □ |

__________________________________________________________________________________

**29 (d). How do you usually get hold of alcohol? (Mark one or more boxes with a cross.)**

□ I don’t drink alcohol

□ From siblings

□ From boy-/girlfriend, friends or friends’ siblings

□ From my parents with their permission

□ From home without permission

□ From another adult (aged 20 or over) who gives it to me

□ From another adult (aged 20 or over) who buys it for me

□ I buy it myself at a restaurant, pub, disco or similar

□ I buy medium-strength beer myself in a shop, petrol station or similar

__________________________________________________________________________________

**29 (e). Do your parents know that you drink alcohol?**

□ No, I don’t drink alcohol

□ No, they don’t know about it

□ Yes, they know about a small part of what I drink

□ Yes, they know about roughly half of what I drink

□ Yes, they know about almost everything that I drink

**30. Have your parents offered you alcohol during the past 12 months?**

□ My parents don’t drink alcohol

□ No, never

□ Yes, I’ve been offered it, but I don’t drink alcohol

□ Yes, I have been allowed to drink from my parents’ glass (sipping)

□ Yes, a glass once or twice

□ Yes, a glass more than once or twice

__________________________________________________________________________________

**31. Do you smoke cigarettes?**

| □ No, have never smoked |  |  |
| --- | --- | --- |
| □ No, have only tried it out  □ No, have smoked but have given up  □ Yes, sometimes (e.g. at parties)  □ Yes, almost every day  □ Yes, every day  ____________________________________________________________________________ |  |  |

**32. Do you smoke e-cigarettes?**

| □ No, have never smoked |  |  |
| --- | --- | --- |
| □ No, have only tried it out  □ No, have smoked but have given up  □ Yes, sometimes (e.g. at parties)  □ Yes, almost every day  □ Yes, every day  ____________________________________________________________________________ |  |  |

**33. Do you use snuff?**

| □ No, have never used snuff |  |  |
| --- | --- | --- |
| □ No, have only tried it out  □ No, have used snuff but have given up  □ Yes, sometimes  □ Yes, almost every day  □ Yes, every day |  |  |

**34. Have you ever been offered drugs to try or to buy? (With drugs we mean e.g. marijuana, hash, spice, amphetamines, ecstasy, cocaine, heroin.)**

□ No □ Yes

__________________________________________________________________________________

**35. Have you ever felt like trying drugs?**

□ No □ Yes

__________________________________________________________________________________

**36. Have you ever tried drugs?**

□ No □ Yes ***If you have answered no, go to question 37***.

__________________________________________________________________________________

**36 (b). Which type or types of drugs have you used? (Mark one or more boxes with a cross.)**

□ Cannabis (hash or marijuana)

□ Spice (or similar smoke mixtures)

□ Amphetamines

□ Cocaine

□ Prescription sedatives/tranquilizers without having a prescription (e.g. Xanax, Stesolid, Zopiclone)

□ Prescription painkillers without having a prescription (e.g. Tramadol, Citodon, Oxycontin)

□ Ecstasy

□ LSD, magic mushrooms or other hallucinogens

□ Heroin

□ Other type, state the type below:

□ Don’t know

State other type ………………………………………………………………………..

__________________________________________________________________________________

**36 (c). How many times during the past 12 months have you used drugs?**

| □ Never | □ 1 time | □ 2-3 times | □ 4-5 times | □ 6-10 times | □ More than 10 times |
| --- | --- | --- | --- | --- | --- |
| *There now follow some more questions on alcohol, tobacco and drugs* | | | | | |

| **37. How true are the following statements for your parents? (Answer for the parent/parents that you live with most.)** | Not true at all | | Fairly untrue | Neither true nor untrue | Fairly true | Very true |
| --- | --- | --- | --- | --- | --- | --- |
| My parents think it is okay if I drink alcohol | | □ | □ | □ | □ | □ |
| My parents think it is okay if I drink and become intoxicated | | □ | □ | □ | □ | □ |
| My parents think it is okay if I smoke cigarettes | | □ | □ | □ | □ | □ |
| My parents think it is okay if I smoke e-cigarettes | | □ | □ | □ | □ | □ |
| My parents think it is okay if I use snuff | | □ | □ | □ | □ | □ |
| My parents think it is okay if I smoke marijuana/hash | | □ | □ | □ | □ | □ |

**38. How big a risk do you think there is that people will harm themselves physically or in some other way, if they…**

|  | No risk | Small risk | Medium risk | Big risk |
| --- | --- | --- | --- | --- |
| …smoke 10 or more cigarettes per day | □ | □ | □ | □ |
| …get intoxicated on alcohol every weekend | □ | □ | □ | □ |
| …try marijuana or hash 1-2 times | □ | □ | □ | □ |
| …use marijuana or hash every weekend | □ | □ | □ | □ |
| …try heroin 1-2 times | □ | □ | □ | □ |

**39. Do you agree or disagree with the following statements?**

|  | Completely untrue | Fairly untrue | Fairly true | Completely true |
| --- | --- | --- | --- | --- |
| If you drink alcohol in your youth, there is a greater risk of becoming addicted to alcohol as an adult | □ | □ | □ | □ |
| It is good that using marijuana or hash is forbidden (illegal) | □ | □ | □ | □ |
| Parents who offer their children alcohol are irresponsible | □ | □ | □ | □ |

| *There now follow some questions about crime* |
| --- |

**40. How many times during the past 12 months have you…**

|  | Never | 1 time | 2-3 times | 4-5 times | 6-10 times | More than 10 times |
| --- | --- | --- | --- | --- | --- | --- |
| Stolen something from a shop? | □ | □ | □ | □ | □ | □ |
| Stolen something from someone’s pocket or bag? | □ | □ | □ | □ | □ | □ |
| Written or painted illegal graffiti? | □ | □ | □ | □ | □ | □ |
| Destroyed something that didn’t belong to you (e.g. broken a window, scratched the paint on a car)? | □ | □ | □ | □ | □ | □ |
| Stolen a bicycle? | □ | □ | □ | □ | □ | □ |
| Taken money or other valuables from someone using threats or violence? | □ | □ | □ | □ | □ | □ |
| Hit someone so that he/she got hurt? | □ | □ | □ | □ | □ | □ |
| Had a knife with you (as a weapon) when you went out? | □ | □ | □ | □ | □ | □ |
| Stolen something from a car or broken into a car in order to steal something? | □ | □ | □ | □ | □ | □ |

| *It is now almost the end of the survey,*  *but first we have a few questions about you* |
| --- |

**41. Mark with a cross whether the following statements are true or not for you!**

|  | Completely untrue | | Fairly untrue | Fairly true | Completely true |
| --- | --- | --- | --- | --- | --- |
| I often do things without stopping to think. | | □ | □ | □ | □ |
| I always try to avoid school assignments that I know are difficult. | | □ | □ | □ | □ |
| I get angry quite easily. | | □ | □ | □ | □ |
| I sometimes take risks just because it is exciting. | | □ | □ | □ | □ |
| I never think about what is going to happen to me in the future. | | □ | □ | □ | □ |
| I sometimes think it’s exciting to do things that can get you into trouble. | | □ | □ | □ | □ |

**42. Mark with a cross the alternative that is most true for you!**

|  | Completely untrue | Fairly untrue | Fairly true | Completely true |
| --- | --- | --- | --- | --- |
| I often feel sad and low. | □ | □ | □ | □ |
| I often worry about the future. | □ | □ | □ | □ |
| I often feel anxious and worried. | □ | □ | □ | □ |
| I often get stomach aches or headaches. | □ | □ | □ | □ |
| I often feel lonely. | □ | □ | □ | □ |
| I have difficulty sleeping and eating. | □ | □ | □ | □ |

**43. Mark with a cross the alternative that is most true for you!**

|  | Completely untrue | Fairly untrue | Fairly true | Completely true |
| --- | --- | --- | --- | --- |
| I have a positive image of myself. | □ | □ | □ | □ |
| I can do anything at all just as well as others. | □ | □ | □ | □ |
| On the whole, I am satisfied with myself. | □ | □ | □ | □ |
| People like me do not have a good chance of succeeding in life. | □ | □ | □ | □ |
| I don’t think I can do anything properly. | □ | □ | □ | □ |
| I haven’t done many good things in my life. | □ | □ | □ | □ |

**What did it feel like to answer these questions? (You can mark more than one box.)**

□ Boring □ Difficult sometimes □ Completely okay

□ Meaningless □ Fun □ Fairly easy

□ Difficult □ Interesting □ Neither fun nor boring

__________________________________________________________________________________

**Here you can write any other comments or thoughts you may have.**

…………………………………………………………………………………………………………………………………………………………….

…………………………………………………………………………………………………………………………………………………………….

…………………………………………………………………………………………………………………………………………………………….

Thank-you for your participation!
